# Mitosis, double strand break repair, and telomeres: A view from the end

**DOI:** 10.1002/bies.201400104

**Published:** 2014-08-29

**Authors:** Anthony J Cesare

**Affiliations:** 1) Children's Medical Research Institute, Genome Integrity GroupWestmead, NSW, Australia; 2) University of Sydney, Sydney Medical SchoolSydney, NSW, Australia

**Keywords:** cell cycle, DNA damage response, double strand break repair, mitosis, telomeres

## Abstract

Double strand break (DSB) repair is suppressed during mitosis because RNF8 and downstream DNA damage response (DDR) factors, including 53BP1, do not localize to mitotic chromatin. Discovery of the mitotic kinase-dependent mechanism that inhibits DSB repair during cell division was recently reported. It was shown that restoring mitotic DSB repair was detrimental, resulting in repair dependent genome instability and covalent telomere fusions. The telomere DDR that occurs naturally during cellular aging and in cancer is known to be refractory to G2/M checkpoint activation. Such DDR-positive telomeres, and those that occur as part of the telomere-dependent prolonged mitotic arrest checkpoint, normally pass through mitosis without covalent ligation, but result in cell growth arrest in G1 phase. The discovery that suppressing DSB repair during mitosis may function primarily to protect DDR-positive telomeres from fusing during cell division reinforces the unique cooperation between telomeres and the DDR to mediate tumor suppression.

## Introduction

Genome integrity is constantly threatened by DNA lesions resulting from both endogenous stress and exogenous insult. Of the many types of genomic damage, double strand breaks (DSBs) present the greatest threat to genomic health. Left unrepaired, a DSB can result in the loss of substantial genetic material. Alternatively, if DSBs are repaired incorrectly this may result in chromosomal structural abnormalities; including dicentric chromosomes that drive further genome instability through a breakage-fusion-bridge cycle. Eukaryotic cells have thus evolved a sophisticated DNA damage response (DDR) that controls DNA repair and cell cycle arrest to cope with the dangers presented by genotoxic stress 1.

While it is critical that cells engage in efficient DSB repair, it is just as important that DSB repair is silenced under certain conditions. One such condition is at the naturally occurring chromosome ends where DSB repair is prevented by specialized nucleoprotein structures called telomeres 2. Another such condition, as shown by the recent discoveries made by Orthwein et al. 3 and Lee et al. 4, is during mitosis. Unlike interphase, when DSB repair prevents genome instability, activating DSB repair during mitosis promotes genome instability 3,4. Surprisingly, DSB repair-dependent genome instability during mitosis results from covalent ligation of chromosome ends 3. The mechanism of silencing DSB repair during mitosis, and at interphase telomeres, is exerted by blocking downstream ubiquitin signaling in the DDR after initial upstream phosphorylation signaling occurs 3–8. Discussed here are recent discoveries related to DDR activation at telomeres and DSB repair silencing during mitosis, with a specific focus on how these activities are intertwined to enable telomere-dependent mechanisms of proliferative arrest and tumor suppression.

## Phosphorylation and ubiquitination regulate DSB repair

**Figure 1:**
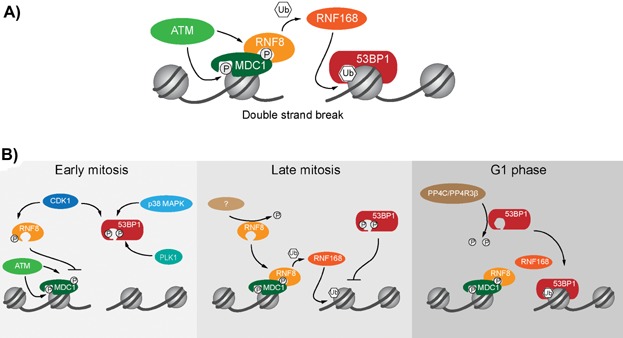
Phosphorylation and ubiquitination regulate DSB repair. A: An abridged representation of the phosphorylation and ubiquitination events at a DSB that recruit 53BP1 and engage NHEJ. B: Depiction of the kinase signaling mechanisms that suppress DSB repair during mitosis and how these signals are reversed as mitotic DSBs transit through cell division into G1 phase.

Spatiotemporal localization of DDR factors during DSB repair is routinely analyzed through observation of cytological ionizing radiation induced foci (IRIF, Fig. 1). In brief, following genomic insult the MRE11/RAD50/NBS1 complex senses a DSB within seconds and then activates ATM at the IRIF 9,10. ATM then phosphorylates histone H2AX on ser139 (γ-H2AX when phosphorylated) in the DSB adjacent chromatin 11. The MDC1 protein binds γ-H2AX 12 and is phosphorylated by ATM, which recruits the RNF8 E3 ubiquitin ligase through direct interaction with phospho-MDC1 13–15. RNF8-dependent ubiquitination recruits another E3 ubiquitin ligase, RNF168, to propagate further ubiquitination at the IRIF 16,17. RNF168-dependent ubiquitination enables 53BP1 to localize to the IRIF 18, and cell-cycle dependent antagonism between BRCA1, 53BP1 and other factors determines if DSB repair proceeds by 53BP1-dependent non-homologous end joining (NHEJ) in G1 phase or BRCA1-dependent homologous recombination in G2 phase 19–22. (The complex molecular processes that occur at DSB repair foci are reviewed in detail elsewhere 23–26.)

In addition to regulating DSB repair, the DDR also controls interphase cell cycle arrest in response to genotoxic stress 1. However, once cells have entered late prophase they are committed to finishing cell division and will not arrest in mitosis as a result of DDR activation 27–29.

## The DDR is dampened during mitosis

While there were indications that the DDR differed in mitotic and interphase cells 30,31, Giunta et al. 5 were the first to present in clear detail the distinctions between mitotic and interphase IRIF. They demonstrated that DSBs induced in metaphase result in upstream ATM activation and IRIF containing γ-H2AX, NBS1 and MDC1, but not RNF8, RNF168, ubiquitin, BRCA1 or 53BP1. Moreover, inducing DSBs in metaphase resulted in muted ATM-dependent DDR checkpoint signaling where ATM was phosphorylated but some downstream ATM targets, including the CHK2 effector kinase, were not 5,31. As damaged cells exited metaphase RNF8 and RNF168 localized to IRIF in late mitosis (anaphase/telophase) 32. Followed by 53BP1 localizing to IRIF in G1 phase, at which time full activation of ATM-dependent checkpoint signaling was engaged 5. The significance of this finding was not properly understood until the molecular mechanism underlying DSB repair silencing during mitosis was recently discovered by Orthwein et al. 3 with contributions made in an independent study by Lee et al. 4.

## Phosphorylation prevents RNF8 and 53BP1 chromatin association during mitosis

Orthwein et al. 3 discovered that RNF8 does not localize to mitotic chromatin because it is phosphorylated on T198 by the mitotic kinase CDK1, which abrogates the physical interaction between RNF8 and MDC1 (Fig. 1). This was confirmed by expressing a phosphomimetic RNF8 T198E protein that did not localize to interphase or mitotic DSBs. Expression of an alanine substitution allele resistant to CDK1 phosphorylation conversely enabled RNF8 T198A to efficiently localize to mitotic IRIF, thus confirming exclusion of RNF8 from mitotic DSB repair was due to CDK1 phosphorylation. As a consequence of RNF8 T198A expression, BRCA1, but not 53BP1, localized to mitotic IRIF. A second mechanism independent of RNF8 therefore remained active to prevent 53BP1 from localizing to mitotic DSBs.

Like RNF8 pT198, 53BP1 pT1609 and pS1618 are residues phosphorylated specifically during mitosis 33, with S1618 defined as a PLK1 target 34. Lee et al. 4 and Orthwein et al. 3 converged on phosphorylation of these residues as the mechanism excluding 53BP1 from localizing to mitotic IRIF (Fig. 1). Both groups identified CDK1 or p38/MAPK as the putative kinases responsible for T1609 phosphorylation and confirmed PLK1-dependent phosphorylation of S1618. Lee et al. 4 further showed that reversal of pT1609 and pS1618 by the PP4C/PP4R3β phosphatase complex enabled 53BP1 localization to G1 phase IRIF.

Both studies found that generating a double phosphomimetic allele (53BP1 T1609E S1618E or “53BP1 TESE”) resulted in the exclusion of 53BP1 TESE from mitotic and interphase IRIF 3,4. T1609 and S1618 are in the 53BP1 ubiquitin-dependent recruitment (UDR) motif. The UDR motif enables 53BP1 to interact with H2A K15-ubiqitin 18, a chromatin mark made by RNF168 35. In vitro experiments demonstrated 53BP1 TESE fragments were unable to bind to H2A K15 ubiquitinated nucleosome core particles 3,4. Lee and coworkers demonstrated that a double alanine substitution phosphorylation insensitive (53BP1 T1609A S1618A or “53BP1 TASA”) 53BP1 TASA protein localized to late mitosis IRIF with endogenous RNF8 and RNF168 4,32, while Orthwein et al. 3 demonstrated that co-expression of 53BP1 TASA and RNF8 T198A fully reconstituted mitotic DSB repair.

## Mitotic DSB repair results in genome instability due to telomere fusions

Mitotic cells are sensitive to DSBs, presumably because DSB repair is silenced 5. However, co-expression of RNF8 T198A and 53BP1 TASA further sensitized mitotic cells to irradiation due to the induction of DSB repair-dependent genomic instability 3. This was evidenced by an increase in kinetochore positive micronuclei, consistent with whole chromosome segregation errors 3. However, the most surprising outcome of restoring DSB repair during metaphase was that the resulting genomic instability stemmed primarily from telomere fusion events 3. Moreover, telomere fusions became more common following irradiation of mitotic cells and this increase in telomere fusions could be suppressed using Hesperadin, an Aurora B kinase inhibitor 3.

Discussed below is the current understanding of how the telomeres cooperate normally with the DDR to mediate cell cycle arrest and the implications of the findings by Orthwein et al. on telomere-dependent tumor suppression.

## Telomeres adopt distinct protective states

**Figure 2:**
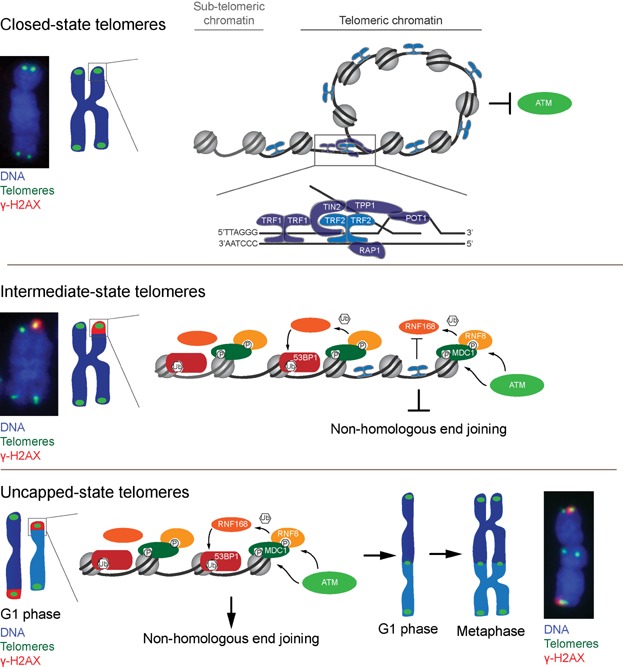
Telomeres prevent DDR activation and chromosome end-joining through distinct protective states. Depicted is a diagrammatic representation of the proposed DDR activity that occurs at a chromosome end in conjunction with each telomere state. Examples of chromosomes from metaphase-TIF assays are shown. Top: Closed-state telomeres are predicted to suppress DDR activity by sequestering the chromosome end and preventing it from activating ATM. The entire six-subunit shelterin complex is shown bound to the t-loop junction in the expanded region. For simplicity only the TRF2 dimer is depicted for the remainder of the shelterin complexes. Middle: Chromosome ends are exposed at intermediate-state telomeres, which allows ATM activation. However, TRF2 retention on the telomeric DNA suppresses NHEJ via the TRF2 iDDR motif, which inhibits RNF168 ubiquitination. 53BP1 is predicted to localize to proximal telomeric or sub-telomeric regions without bound TRF2. Bottom: Uncapped-state telomeres occur when insufficient TRF2 remains bound to the telomere allowing 53BP1 loading at the distal chromosome end and NHEJ activity. After NHEJ occurs the telomere DDR at the fused chromosome end is resolved 6. Chromosome fusions at telomeres typically occur in G1 phase and are easiest to visualize in the ensuing metaphase. The metaphase-TIF assay example on the right shows an uncapped-state telomere that fused in G1-phase and intermediate-state telomeres at the opposed chromosome ends that remained fusion resistant.

DDR activity is modulated at chromosome ends by the telomere-specific “shelterin” protein complex 36,37 (Fig. 2). Within shelterin, TRF2 is the subunit most responsible for inhibiting NHEJ 38,39. Conditional deletion of TRF2 results in a striking phenotype where the DDR is activated specifically at chromosome ends and essentially all telomeres become covalently ligated, resulting in long chains of end-to-end chromosome fusions 40 (Fig. 2). Cytological association of telomeres and DDR factors in these experiments are termed “telomere dysfunction induced foci” (TIF) 41, which are similar to IRIF except they result from altered telomere protection and not genomic damage. Using conditional genetic deletion, or other related experimental systems, it has been comprehensively determined that the DDR controls NHEJ-dependent telomere fusion in the absence of functional TRF2 6,19,36,38,40,42–49.

However, an emerging theme in telomere biology is that telomere deprotection under physiological or pathological conditions is different from telomere dysfunction induced by genetically deleting shelterin subunits 50. Much of this research was performed in the Reddel and Karlseder laboratories and was enabled by automated imaging of telomere deprotection in cyto-centrifuged prometaphase or metaphase spreads (i.e. the metaphase-TIF assay) 7. This approach revealed quantitative and structural information pertaining to telomere protection that was hidden in interphase nuclei.

We now understand that human telomeres adopt three distinct protective states in relation to DDR activation and DSB repair 7,50 (Fig. 2). “Closed-state” telomeres suppress DDR activation by adopting a protective structure that hides the chromosome end from recognition by the DDR. It has been hypothesized that telomere loops (t-loops) 51,52 are the protective structure sequestering the distal chromosome end from DDR activation. Failure to form the closed-state structure results in “intermediate-state” telomeres that activate an ATM-dependent DDR, but where NHEJ remains suppressed if some TRF2 remains bound to the DDR-positive telomeric chromatin. Chromosome end-to-end fusions only occur at fully “uncapped-state” telomeres that lack sufficient TRF2 to inhibit DSB repair. TRF2 deletion therefore directly transitions telomeres from closed- to uncapped-state 40,42. Whereas a partial TRF2 depletion was recently shown to induce intermediate-state telomeres, while a more extensive TRF2 depletion was required to induce both intermediate- and uncapped-state telomeres 6.

## The DDR at mitotic DSBs and intermediate-state telomeres is similar

Observation of spontaneous intermediate-state telomeres in immortalized or cancer cells indicated that TRF2 possessed independent protective functions which (1) inhibited DDR activation and (2) suppressed NHEJ 7,53. This was supported by subsequent findings showing that DSBs induced within telomeric DNA are not repaired 54,55, presumably because TRF2 inhibits NHEJ at telomeric DSBs. The mechanism underlying the independent protective functions of TRF2 was recently discovered by the Lazzerini Denchi lab, when they found that TRF2 silences ATM-activation via its dimerization domain independently from suppressing NHEJ via its iDDR motif 8. TRF2-dependent NHEJ silencing through the iDDR motif is exerted by preventing RNF168 ubiquitination after initial DDR activation, reminiscent of how ubiquitination is suppressed during mitotic DSB repair. A fascinating related observation is that intermediate-state telomeres are bound by 53BP1 during interphase 6. This most likely represents γ-H2AX spreading at intermediate-state telomeres to regions proximal to bound TRF2 56, allowing RNF168 ubiquitination and 53BP1 association with proximal telomeric or sub-telomeric chromatin without engaging telomere NHEJ. If this interpretation is correct it suggests 53BP1 must be spatially located adjacent to the DNA end for NHEJ to occur.

Also reminiscent of the mitotic DDR, intermediate-state telomeres in asynchronous cultures induce muted ATM checkpoint signaling where ATM is activated but CHK2 and NBS1 are not phosphorylated 6. Full activation of ATM-dependent DDR signaling occurs in response to uncapped-state telomeres when NHEJ is ongoing or has already occurred. This is similar to when DSBs are induced in mitosis and ATM signaling is muted until the cells enter G1 phase and DSB repair is engaged 5,31.

## Intermediate-state telomeres pass through cell division prior to growth arrest

In the recent study using TRF2 knockdown to induce intermediate- and uncapped-state telomeres it was observed that the telomere DDR is refractory to G2/M checkpoint activation 6. Both fixed and live cell imaging revealed DDR-positive telomeres in TRF2 shRNA treated cells were present in S and G2 phase, and that γ-H2AX labeled telomeres pass through mitosis as epigenetic marks into the G1 phase daughter cells. Inheriting intermediate-state telomeres from the previous cell division resulted in G1 phase cell cycle arrest in p53 competent primary fibroblasts 6. Moreover, observations of spontaneous telomere deprotection in aged human cells are consistent with p53-dependent G1 phase replicative senescence being induced after a threshold of five intermediate-state telomeres are inherited from the previous mitosis 53,57. p21, a transcriptional target of p53, was recently shown to control a mechanism where growth restriction at G0 was decided in the previous G2 phase 58, reminiscent of what is observed during telomere DDR dependent growth arrest.

## 53BP1 dissociates from deprotected telomeres at G2/M and re-associates in G1 phase

The initial static observations of spontaneous telomere deprotection revealed that γ-H2AX, MRE11 and MDC1, but not 53BP1, localized to metaphase-TIF (7, Cesare and Reddel unpublished). Recent dynamic visualization of telomere deprotection in TRF2 depleted cells revealed that 53BP1 bound to TIF in G2 phase dissociates from the telomeric chromatin at the G2/M boundary and then re-associates with TIF in the G1 phase daughter cells after they pass through mitosis 6. 53BP1 dissociation from TIF at G2/M therefore suggests the mechanisms described above not only prevent RNF8 and 53BP1 from associating with DSBs during cell division, but that mitotic kinases may also function to actively remove RNF8 and 53BP1 from DDR foci before cells enter mitosis.

## The telomere DDR-dependent prolonged mitotic arrest checkpoint

Another surprising recent finding related to telomeres, mitosis, and the DDR was the discovery of the telomere-dependent prolonged mitotic arrest checkpoint 59. In this study, Hayashi et al. found that when human cells are held in a prolonged mitotic arrest of around six hours or more, this resulted in an ATM-dependent telomere DDR. The telomere DDR during prolonged mitosis was determined to be Aurora B dependent, because it could be suppressed by treating cultures with Hesperadin before mitotic entry. Hesperadin treatment therefore allowed a distinction to be made between TIF that were carried into mitosis from the previous G2 phase, which are Hesperadin insensitive, and those that arise due to prolonged mitotic arrest, which are Hesperadin sensitive 6.

The telomere DDR during prolonged mitosis was shown to result from a partial TRF2 dissociation from the telomeric DNA 59. After being released from a prolonged mitotic arrest, the DDR-positive telomeres passed into the G1 phase daughter cells, which induced p53-dependent growth arrest without CHK2 phosphorylation, consistent with the differential ATM signaling induced by intermediate-state telomeres 6,59. Chromosome fusions were not observed as a result of the prolonged mitotic arrest, even when combined with partial TRF2 depletion and release into G1 phase, further indicative that prolonged mitotic arrest induces intermediate-state telomeres 6,59.

## Do intermediate-state telomeres fuse during mitosis if DSB repair is derepressed?

**Figure 3:**
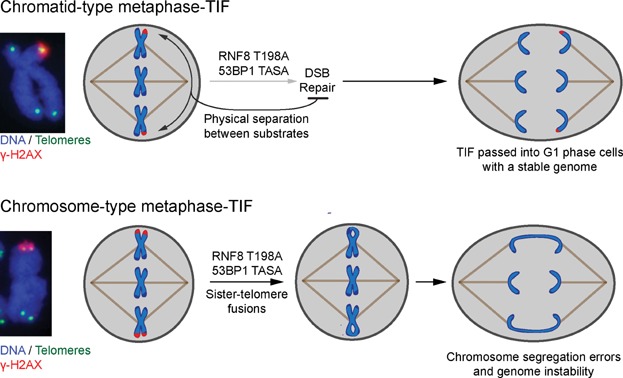
Putative mechanism for sister-telomere fusion in RNF8 T198A and 53BP1 TASA expressing cells. Examples of chromatid- and chromosome-type metaphase TIF are shown on the left. Chromatid-type metaphase-TIF are likely resistant to fusion due to physical separation in mitotic cells. Whereas, chromosome-type metaphase-TIF appear to fuse during mitosis in RNF8 T198A and 53BP1 TASA expressing cells, which results in sister-telomere fusions, chromosome segregation errors and genomic instability.

It is not clear why telomeres fused when DSB repair was activated during mitosis. However, a likely answer is that intermediate-state telomeres, which normally suppress NHEJ during interphase, fuse during cell division in DSB repair competent mitotic cells (i.e. in a RNF8 T198A and 53BP1 TASA over-expression background) (Fig. 3). For DSB repair to occur there must first be a DDR, and metaphase-TIF are the most predominant form of spontaneous DDR foci in mitotic cells 7,53,60. In aged and cancer cells spontaneous metaphase-TIF are present in distinct chromatid (γ-H2AX on one telomere sister-chromatid) and chromosome (γ-H2AX on both telomere sister-chromatids) types, with chromatid-type being the more abundant form 7,53. Almost all the telomere fusion events observed by Orthwein et al. 3 were between telomere sister-chromatids on the same metaphase chromosome end; indicating that telomere fusions were mostly limited to ligation of sister-chromatid telomeres at chromosome-type metaphase-TIF and that fusion did not readily occur between chromatid-type metaphase-TIF at different metaphase chromosome ends. This may indicate mitotic telomere fusions, unlike telomere fusions during interphase 46, are limited to substrates in close spatial proximity. Supporting these predictions, the numbers of sister-telomere fusions observed by Orthwein et al. in DSB repair competent mitotic cells in the absence of irradiation are consistent with the abundance of spontaneous chromosome-type metaphase-TIF previously observed in the cell types they used 3,6.

Why intermediate-state telomeres fuse when mitotic DSB repair is activated is not immediately clear, as some TRF2 remains bound at metaphase-TIF in aged cells and during prolonged mitotic arrest 6,53. It is possible that chromatin compaction during mitosis places 53BP1 TASA, which is likely bound to proximal telomeric or sub-telomeric regions at intermediate-state telomeres, adjacent to the chromosome end in three dimensional space. This may facilitate NHEJ at the TRF2 bound telomeres. Alternatively, the single-strand 3′ telomeric DNA overhang, which normally assists in preventing end fusions 61–64, is removed during prolonged mitotic arrest 59. If overhangs are removed from metaphase-TIF as a general phenomenon this may also contribute to telomere fusions in DSB repair competent mitotic cells.

## Is mitotic DSB repair suppressed to mediate telomere-dependent tumor suppression?

The passage of intermediate-state telomeres through mitosis is likely a critical feature of telomere-dependent tumor suppression 6,50. Due to the properties of progressive telomere erosion during cellular aging, shortened intermediate-state telomeres occur before chromosome ends lose the entirety of their telomeric DNA and become uncapped-state 53. Intermediate-state telomeres have been shown to arise spontaneously in aged cells during S or G2 phase 7,53. This was proposed to result from a failure to reform the closed-state after it is opened by DNA replication 7,50,65–67. By allowing intermediate-state telomeres to pass through G2 and mitosis, it ensures a stable G1 phase growth arrest in diploid cells at senescence before excessive telomere shortening can induce uncapped-state telomeres and genome instability. Viewed in this light, the DDR at intermediate-state telomeres is a normal biological outcome of cellular aging that promotes telomere-dependent growth arrest and tumor-suppressive senescence while preventing telomere fusions and genomic instability.

Intermediate-state telomeres routinely pass through mitosis in increasing abundance during cellular aging, until sufficient numbers accumulate to induce senescence 53,57. If DSB repair was active during mitosis the transit of intermediate-state telomeres through cell division would drive genome instability in pre-senescent cells. It therefore appears essential that “mitosis inhibits DNA double-strand break repair to guard against telomere fusions” as ascertained by Orthwein et al. 3.

## Why do genomic DSBs induced during mitosis cause telomere fusions?

When DSB repair competent mitotic cells were irradiated to induce non-specific genome wide damage, this resulted in telomere-fusion-dependent genomic instability 3. Moreover, Orthwein et al. 3 found irradiation of DSB repair competent mitotic cells resulted in increased metaphase-TIF and greater numbers of sister-telomere fusions, both of which were Hesperadin sensitive. This suggests the telomere DDR in mitotic cells following genome wide damage is connected to the prolonged mitotic arrest checkpoint and not a result of DSBs induced in the telomeric DNA. How then does non-specific genomic damage cause an Aurora B dependent telomere DDR and sister-telomere fusions?

A potential answer is that Aurora B activation of the telomere DDR during prolonged mitosis is a general response to accumulating stress in mitotic cells. This is supported by the observation that increasing duration of mitotic arrest corresponds with increasing numbers of metaphase-TIF 59. Irradiating mitotic cells will amplify cellular stress, potentially accelerating the Aurora B-dependent telomere DDR. This would in turn result in more metaphase-TIF. Which in DSB repair competent mitotic cells would result in more sister-telomere fusions despite the initial induction of non-specific genome wide damage.

## Conclusions

The recent discoveries described here highlight our emerging understanding of the complex relationship between telomeres, mitosis, and the DDR. Suppressing DSB repair during mitosis appears to ensure that intermediate-state telomeres pass through cell division to facilitate telomere-dependent growth arrest in stable diploid G1 phase cells without inducing genomic instability. However, much about the connectivity between mitosis, DSB repair and telomeres remains to be understood. For example, what is the molecular mechanism that facilitates telomere fusion in DSB repair competent mitotic cells? Also, what is the mechanism linking genomic breaks induced during mitosis to the Aurora B dependent mitotic telomere DDR, and how does this impact cells with silenced mitotic DSB repair? It is also unclear if 53BP1 dissociation from TIF at the G2/M boundary is controlled by the same mechanism that prevents 53BP1 from associating with mitotic IRIF or if there is another independent level of control regulating mitotic DSB repair that remains to be discovered.

An appealing prospect raised by the discovery of the mechanism inhibiting mitotic DSB repair is that the spontaneous occurrence of abundant intermediate-state telomeres in many cancer cells may present a vulnerability to be exploited for therapeutic means. A combinatorial approach that arrests cancer cells in mitosis while simultaneously targeting mitotic kinases and/or the DDR may hold promise to induce telomere fusions that kill tumor cells. It will be exciting to unravel this surprising and complex relationship between telomeres, mitosis, and DSB repair and to identify the implications of this relationship on human aging and oncogenesis.

## Abbreviations

DDR: DNA damage response
DSB: double strand break
IRIF: ionizing radiation induced foci
NHEJ: non-homologous end joining
TIF: telomere dysfunction induced foci
t-loop: telomere loop
